# Aerosol infection of Balb/c mice with eastern equine encephalitis virus; susceptibility and lethality

**DOI:** 10.1186/s12985-018-1103-7

**Published:** 2019-01-05

**Authors:** Amanda L. Phelps, Lyn M. O’Brien, Lin S. Eastaugh, Carwyn Davies, Mark S. Lever, Jane Ennis, Larry Zeitlin, Alejandro Nunez, David O. Ulaeto

**Affiliations:** 1 CBR Division, Defence Science and Technology Laboratory (Dstl), Room 201, Building 7a,, Porton Down, Salisbury, Wiltshire SP4 0JQ UK; 2 Mapp Biopharmaceutical Inc, 6160 Lusk Blvd. #C105, San Diego, CA 92121 USA; 3 Pathology Unit, Animal and Plant Health Agency – Weybridge, Woodham Lane, New Haw, Addlestone, Surrey KT15 3NB UK

**Keywords:** Eastern equine encephalitis virus, EEEV, Alphavirus, Pathogenicity, MLD, Mouse, Model, Aerosol

## Abstract

**Background:**

Eastern equine encephalitis virus is an alphavirus that naturally cycles between mosquitoes and birds or rodents in Eastern States of the US. Equine infection occurs by being bitten by cross-feeding mosquitoes, with a case fatality rate of up to 75% in humans during epizootic outbreaks. There are no licensed medical countermeasures, and with an anticipated increase in mortality when exposed by the aerosol route based on anecdotal human data and experimental animal data, it is important to understand the pathogenesis of this disease in pursuit of treatment options. This report details the clinical and pathological findings of mice infected with EEEV by the aerosol route, and use as a model for EEEV infection in humans.

**Methods:**

Mice were exposed by the aerosol route to a dose range of EEEV to establish the median lethal dose. A pathogenesis study followed whereby mice were exposed to a defined dose of virus and sacrificed at time-points thereafter for histopathological analysis and virology.

**Results:**

Clinical signs of disease appeared within 2 days post challenge, culminating in severe clinical signs within 24 h, neuro-invasion and dose dependent lethality. EEEV was first detected in the lung 1 day post challenge, and by day 3 peak viral titres were observed in the brain, spleen and blood, corresponding with severe meningoencephalitis, indicative of encephalitic disease. Lethality follows severe neurological signs, and may be linked to a threshold level of virus replication in the brain. Effective medical countermeasures for EEEV may necessitate early inoculation to inhibit infection of the brain in zoonotic incidents, and be able to traverse the blood-brain barrier to sufficiently interrupt replication in the brain in cases of aerosol infection.

**Conclusions:**

There is little human data on the hazard posed by aerosol infection with encephalitic alphaviruses, and use of EEEV as a bioweapon may be by the aerosol route. A well characterized model of aerosol exposure that recapitulates some of the most severe human clinical features is necessary to evaluate the efficacy of putative medical countermeasures, and to increase our understanding about how this route of infection induces such rapid neuro-invasion and resulting disease.

## Background

Alphaviruses are arthropod-borne, positive sense single-stranded RNA viruses of the family *Togaviridae,* comprising of a number of viruses that are pathogenic for animals and humans. There are four antigenic subtypes of Eastern equine encephalitis virus, one that circulates in North America (EEEV), and three that circulate in Central and South America (recently reclassified as a new species, designated Madariaga viruses; ICTV 9th edition). The Madariaga viruses rarely cause disease in humans, and are usually associated with a minor to severe arthritic disease. EEEV causes a mild to severe encephalitis in both equines and humans, resulting in an average of 7 neuro-invasive disease cases a year in humans in the US (CDC, [1]). EEEV is maintained in a sylvatic cycle between mosquitos and birds or rodents. Transmission to equines occurs via cross-feeding mosquitoes, and human infections generally occur in proximity to equine outbreaks. Human infections are generally asymptomatic but may result in the sudden onset of mild flu-like symptoms; fever, malaise, chills, arthralgia and myalgia. In a minority of cases neuro-invasive disease develops, typically characterised by fever, headache, irritability, restlessness, vomiting, convulsions and encephalitis, potentially leading to coma and death. The human mortality rate of neuro-invasive disease is reported to be approximately 36–75% [2], and up to 90% of survivors of neuro-invasive EEEV disease are left with incapacitating and progressive neurological sequelae, that require significant healthcare intervention [3]. Many people with severe sequelae die within a few years of initial EEEV infection. The National Institute of Allergy and Infectious Diseases, and the Centre for Disease Control list EEEV as a Category B threat agent, in part due to the high mortality rate, high infectivity rate, and its potential use as a biological warfare (BW) agent. Additionally, there are no medical countermeasures for the treatment of EEEV disease, patients receive supportive therapy only.

Of the three encephalitic New world alphaviruses (EEEV, Western equine encephalitis virus and Venezuelan equine encephalitis virus), EEEV is the least well studied and understood, with limited publications pertaining to animal models and pathogenesis of disease. Mice are susceptible to EEEV infection by intra-cranial, sub-cutaneous, intra-peritoneal and aerosol routes of infection, with reported LD_50_ (dose required to cause 50% lethality) values of ~ 1, 1250, 400, 500 plaque forming units (pfu) respectively [4, 5]. Clinical observations have been made of seizures but not paralysis in EEEV infected mice, which is also true of Western equine encephalitis virus (WEEV) infected mice exposed by the aerosol route of infection [6]. Venezuelan equine encephalitis virus (VEEV) infected mice exposed by the aerosol route however, do not present with seizures, but paralysis is evident. In recent studies, adult Balb/c mice were exposed to EEEV strain FL93–939 by the aerosol (whole body exposure), intra-nasal and sub-cutaneous routes of infection, fully characterizing the clinical course of disease and pathogenesis of this strain [7, 8]. The severity, lethality and onset of disease is route dependent. Mice exposed by the aerosol route developed clinical signs of disease 3 days post exposure and were found to have EEEV in the brain from 6 h post exposure. Aerosol exposure was by way of whole body bioaerosol exposure system, allowing free movement of animals within the exposure cage, but with the potential to deposit viable virus on mucosal surfaces and fur. An alternative approach is a nose-only aerosol exposure, which does not have this potential, but does mean the animals are physically restrained for the duration of the exposure. The intra-nasal route of infection provided similar findings to the aerosol route, albeit in a delayed manner, with both routes of exposure culminating in 100% mortality. The sub-cutaneous route of infection was not as severe regarding overt clinical signs of disease, fever or weight loss, and virus titres in the brain were low or absent. The sub-cutaneous route of infection is comparable to a mosquito bite, and like human disease, did not culminate in 100% mortality. The route of infection is clearly an important mortality factor and exposure of humans to EEEV by the aerosol route is likely to increase the case fatality rate beyond what is observed in epizootic outbreaks. There are very little data from human infections on the differential hazard presented by aerosol infection with encephalitic alphaviruses. The available evidence comes from accidental laboratory exposures, which indicate aerosol exposure of humans leads to rapid neuro-invasion and more aggressive disease. The accidental exposure of laboratory personnel to WEEV by the aerosol route increased the case fatality rate to 40%, from 15% by other routes [9]. Because any use of EEEV as a bioweapon is likely to use the aerosol route, a well characterized mouse model of aerosol exposure that provides rapid neuro-invasion is necessary to evaluate the efficacy of medical countermeasures, and also to further understand how this route of infection induces such rapid neuro-invasion and encephalitic disease.

Non-human primate models have also been assessed as a model for human EEEV disease. Old world (rhesus and cynomolgus macaques) and New world monkeys (marmoset) have been reported to appropriately recapitulate the human condition of EEEV disease [10–13]. A non-human primate model of disease that appropriately models the human condition is often sought under the FDA ‘Animal Rule’, in pursuit of licensure for any candidate medical countermeasure where human efficacy trials are not ethical or feasible [14]. However, as they belong to the highest order of mammals, the use of non-human primates has additional ethical considerations, as well as practical considerations for use in experimental facilities. Article 7 of the European Directive 86/609/EEC states: “When an experiment has to be performed, the choice of species shall be carefully considered and, where necessary, explained to the authority. In a choice between experiments, those which use the minimum number of animals, involve animals with the lowest degree of neurophysiological sensitivity, cause the least pain, suffering, distress or lasting harm and which are most likely to provide satisfactory results shall be selected”. As such, the employment of appropriate small rodent models of human disease in the fundamental stages of research is considered a good foundation on which to build a body of evidence for progressive studies in non-human primates.

We present the course of EEEV disease in adult Balb/c mice when exposed to a nose-only aerosol of EEEV strain Pe-6. The Median Lethal Dose (MLD); pathogenesis, including histopathological and immuno-histochemistry findings; and clinical scoring system are reported.

## Methods

### Cells and virus

Vero cells (European Collection of Animal Cell Cultures, UK) were cultured in Dulbecco’s minimal essential medium with 10% (*v*/v) foetal calf serum, 50 IU/ml penicillin, 50 μg/ml streptomycin and 2 mM L-glutamine, in a 5% CO_2_ humidified atmosphere, at 37 °C. During virus culture, Vero cells were maintained in Leibovitz L-15 (L-15) medium supplemented with 2% (v/v) foetal calf serum, 50 IU/ml penicillin, 50 μg/ml streptomycin and 2 mM l-glutamine (Maintenance Media; MM), at 37 °C in a humidified atmosphere without added CO_2_. Aerosol challenge preparations were made in L-15 medium supplemented with 2 mM L-glutamine only (Challenge Media; CM). All media and supplements were sourced from Sigma, UK or Gibco, ThermoFisher Scientific, UK.

Standard plaque assays were performed in a 24-well plate format with 100 μl/well of virus inoculum applied to wells in duplicate, with up to 30 min of gentle rocking applied to plates during adsorption. Plates were incubated at 37 °C in a humidified atmosphere without added CO_2_ for 3 days with 1 mL overlay of carboxymethylcellulose. Monolayers were then fixed with 10% (*v*/v) neutral buffered formalin (NBF) (Sigma, UK) overnight, and then visualized by staining with 0.1% (*w*/*v*) crystal violet in 30% (v/v) ethanol. The limit of detection in this assay is 10 plaque forming units (pfu)/mL of original sample. The exception was titration of impinger samples (output virus) which were performed in a 6-well plate format with 500 μl/well of virus inoculum applied to wells in duplicate. The limit of detection for impinger samples is 2 pfu/mL.

EEEV strain Pe-6 was kindly supplied by Dr. L Nagata from Defense Research and Development, Canada. Challenge virus stocks were prepared by inoculating 2–3 day old Balb/c suckling mouse pups intra-cranially with 10^4^ pfu/10 μL EEEV Pe-6 and allowing them to become moribund (24 h after inoculation) before culling them with an overdose of sodium pentobarbital. Pregnant adult female Balb/c mice were sourced from Charles River Laboratories, UK (see in vivo mouse studies below for further details). Virus was harvested by extracting brain tissue through the dorsal cranium with a large-bore syringe needle and mixed with Leibovitz L-15 medium supplemented with 2% (*v*/v) foetal calf serum. This was then passed through a 70 μm nylon cell strainer (BD Falcon ThermoFisher Scientific, UK), clarified by centrifugation at 10,000 rpm for 10mins in a SW28 rotor (Beckman Coulter, UK), and stored at − 80 °C until required. All work with EEEV was carried out under UK Advisory Committee on Dangerous Pathogens Level 3 (ACDP3, BSL3) containment.

### Aerosolisation of EEEV and calculation of the presented challenge dose

Aerosols were generated using a 3-jet Collison nebulizer, containing a minimum volume of 10 mL of virus suspension in CM (input virus), controlled and conditioned to 50% (± 5%) relative humidity, by an AeroMP platform system (Biaera Technologies, Hagerstown, MD, USA). Aerosols were generated for a total of 9 min, with aerosol sampling achieved using an all-glass impinger (AGI-30; Ace Glass, Vineland, NJ, USA) containing 10 mL phosphate buffered saline (output virus). A total of three samples were taken during each 9 min aerosolisation for 1 min each, at a flow rate of 12 L/min. All virus samples were kept on ice until used or titrated. Once the relationship between input and output virus was established, the aerosol challenge preparations for in vivo studies were generated as described above. A maximum of 20 animals (see below) were exposed in any single aerosol exposure, and were physically restrained in holding tubes, enabling nose-only exposure to the aerosol. A fresh preparation of virus was used for every 20 animals exposed, and a sample of each aerosol exposure was taken using an impinger, as described. The mean calculated, presented challenge dose was determined using the viral titres obtained from the impingers (output virus), and Guyton’s formula for the respiratory volumes of laboratory animals [15].

### In vivo mouse studies

All studies were performed in accordance with the UK Scientific Procedures (Animals) Act 1986 and the UK Codes of Practice for the Housing and Care of Animals Used in Scientific Procedures 1989 and reviewed by the Animal Care and Use Review Office (Fort Detrick, MD, USA). Micro-chipped female, Balb/c mice, aged 7–9 weeks (18–20 g; Charles River Laboratories, Margate, UK), were suitably housed with access to food and water ad libitum in a rigid-walled BSL3 containment isolator. Acclimatization within the BSL3 isolator was for a minimum of 5 days. A clinical scoring system already established for Balb/c mice for two closely related pathogenic alphaviruses, VEEV and WEEV, was used to closely monitor the clinical course of infection [6]. Typically mice may present with observable piloerection (score of 1) and hunched posture (score of 1) before rapidly progressing with more severe clinical signs [6]. Any mouse observed to have pronounced mobility issues (unable to reach food and water) was culled on welfare grounds, and in accordance with UK Home Office Project License requirements. Mice were observed a minimum of twice a day for clinical signs of infection post-challenge, by an independent technician. All culls were performed according to the UK Schedule 1 method (cervical dislocation followed by confirmation of cessation of heart beat).

### Median lethal dose (MLD)

Groups of mice (*n* = 8) were challenged with a dilution range of EEEV Pe-6 by the aerosol route. Virus was diluted in CM to yield 5 × 10^7^ pfu/ml, and 10-fold dilutions were prepared to 5 × 10^2^ pfu/ml for use as input virus in the 3-jet Collison nebulizer. A control group of mice (*n* = 7) was used in the first exposure and challenged with CM only (no virus) by the aerosol route. All mice were individually weighed daily and scored for clinical signs of disease a minimum of twice daily. Statistical analysis was performed using Excel 2010 (Analysis ToolPak), single factor analysis of variance. As mice succumbed to disease, key tissues were excised to determine viral load. Calculation of the MLD was based on the number of surviving animals from each challenge group 14 days post-challenge, using the 50% end point calculation of Reed & Muench [16].

### Pathogenesis study

Mice were challenged by the aerosol route with approximately 10 x MLD presented dose EEEV Pe-6 or with CM alone (no virus), as described. During the incubation period (1–3 days post-challenge), overt disease (4 days post-challenge) and convalescent periods (10 days post-challenge), groups of EEEV challenged mice were culled to make assessments of viral load and distribution, as well as histopathological consequences of infection. A control group of mice exposed to CM alone were culled 1 day post-challenge. At scheduled time-points mice were anaesthetized with gaseous halothane and exsanguinated by cardiac puncture prior to being culled by cervical dislocation. The brain, lung and spleen of these animals were excised to determine viral load. Tissues were homogenised through a 40 μm cell sieve (Corning Falcon cell strainer, Fisher Scientific, UK) into 1 mL MM, and dilutions prepared (also in MM) for a standard 24-well format plaque assay as described above. Neat samples were also used, with the exception of neat blood samples as they obfuscated the cell monolayer, making it difficult to visualise any plaques. The limit of detection for blood samples was therefore higher (100 pfu/ml) than for other tissues (10 pfu/ml). Viral titres (pfu/ml) were adjusted based on the weight of individual tissues (pfu/g).

Additionally, at scheduled time-points, a sub-set of at least 3 whole mice were terminally anaesthetized with gaseous halothane, and prepared for immersion in 10% NBF for histopathological analysis. Carcass preparation required full abdominal, thoracic and cranial cavity penetration of the NBF to ensure complete virus inactivation prior to removal from containment laboratories. A control group of 3 mice challenged with CM only, was included and culled 1 day post-challenge.

For histopathological analysis, tissues were dissected from each individual carcass and routinely processed and embedded in paraffin wax. The head, incorporating the jaw, nasal cavity, nasal/oral mucosa, ocular tissue, and femur were decalcified post fixation in Gooding and Stewart solution (1:1:18 solution of 96–100% formic acid (VWR Chemicals, UK), 36% formaldehyde (VWR Chemicals) and purified water) for a minimum of 48 h prior to embedding. Six coronal sections were prepared from the brain, to permit examination of the olfactory bulbs, cortex at various levels, hippocampus, thalamus, hypothalamus, pons, cerebellum, and brainstem. Cross sections of the head, two at eye level, one 5 mm rostral, and one longitudinal section of femur (post decalcification), as well as lung, liver, spleen, heart, trachea, mediastinal structures including thymus and mediastinal lymph nodes, mandibular and accessory mandibular lymph nodes, and salivary glands were also processed.

Cross sections 4 μm thick were prepared and either stained with haematoxylin and eosin, or used for immuno-histochemical (IHC) detection of EEEV antigen using the novel anti-EEEV 4B3 monoclonal antibody (experimental mAb developed by Dr. W Hu, DRDC, Canada). Virus was detected with the use of automated protocols optimised for use on the Ventana Discovery XT staining module (Ventana Medical Systems, AZ, USA). Formalin-fixed paraffin wax embedded (FFPE) tissue sections were first de-waxed using a standard CC2 (Ventana Medical Systems, AZ, USA) cell conditioning regime followed by Protease 1 (Ventana Medical Systems, AZ, USA) for 16 min. Primary anti-EEEV mAb (10 μg/ml) diluted in Ventana Ab Diluent (Ventana Medical Systems, AZ, USA) was then added for 60 min and incubated at room temperature. The antibody-antigen interaction was amplified using OMap anti-mouse HRP multimer (Ventana Medical Systems, AZ, USA), and then visualised using the ChromoMap DAB kit (Ventana Medical Systems, AZ, USA). FFPE tissue sections were then counterstained in haematoxylin (Ventana Reagents, AZ, USA) for 8 min, prior to permanent mounting for analysis. Concentration-matched mouse (10 μg/ml) antibody isotype (Vector Laboratories Peterborough, UK) controls were diluted in Ventana Ab diluent as negative controls.

## Results

### Aerostability of EEEV Pe-6

**Fig. 1:**
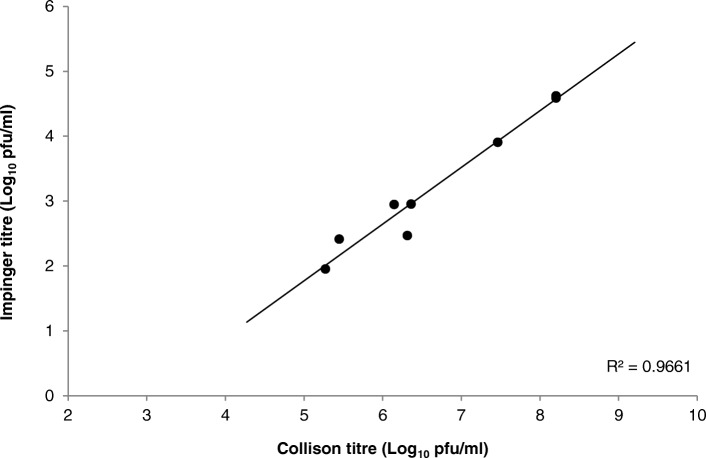
Correlation between input (Collison) and output (Impinger) virus titres of EEEV Pe-6 when aerosolized using an AeroMP platform. *n* = 3 for each of three independent experiments

The ability to expose experimental animals to any aerosolised agent requires prior knowledge of the aerostability of that agent. Under defined laboratory conditions, three independent experiments were conducted to assess the viability of EEEV Pe-6 when aerosolised in the AeroMP exposure system. In each experiment, during a 9 min test exposure, three impinger samples were taken to evaluate the relationship between the input virus (3-jet Collison nebuliser) and output virus (All Glass Impinger-30). A linear relationship was observed, with an average difference of 3.5 Log_10_ pfu/ml between input and output virus titres (Fig. 1). This is consistent with the input and output titres of the closely related alphavirus, WEEV (3.4 Log_10_ pfu/ml), when aerosolised using the same defined laboratory conditions and aerosol apparatus [6].

### Calculation of the median lethal dose (MLD) by the aerosol route

Adult Balb/c mice were exposed nose-only by the aerosol route to a dose range of virus and monitored for 14 days post-challenge. In direct correlation with the titre of input virus in the Collison (Table 1), the mean calculated, presented challenge dose ranged from 2.8 × 10^3^ to < 2 pfu/mouse. The limit of detection for this assay is 2 pfu/ml, which means that virus may have been present in output aerosol samples but in a quantity below the limit of detection.

**Table 1:** Calculated presented challenge dose of EEEV (Pe-6) to groups of female Balb/c mice, with subsequent survival and clinical data. Each aerosol challenge utilised 10 mL of fresh virus preparations, in a 10 mins nose-only aerosol exposure

INPUT Collison (pfu/ml)	OUTPUT Impinger (pfu/ml)	Calc. Presented Dose (pfu/mouse)	Survival (%)	Mean Time to Death (days)	Max. mean weight loss (%) ^a, b^	Max. mean clinical score ^a, b^	Max. viral load in brain (Log_10_ pfu/g)^c^
0.0E+ 00	0.0E+ 00	MOCK	8/8 (100)	–	1.2, n/a	0.0, n/a	–
1.6E+ 02	0.0E+ 00	0.0E+ 00	8/8 (100)	–	0.1, n/a	0.0, n/a	–
1.8E+ 03	6.0E+ 00	1.0E+ 00	8/8 (100)	–	0.6, n/a	0.0, n/a	–
2.3E+ 04	4.4E+ 01	7.0E+ 00	8/8 (100)	–	1.0, n/a	0.0, n/a	–
1.9E+ 05	1.4E+ 02	2.4E+ 01	7/8 (88)	3.1	2.0, 11.6	0.5, 4.0	9.9 (*n* = 1)
3.1E+ 06	2.5E+ 03	4.3E+ 02	3/8 (38)	4.2	6.4, 13.3	3.3, 8.0	9.7 (*n* = 3)
2.0E+ 08	1.7E+ 04	2.8E+ 03	0/8 (0)	3.0	12.9, 12.9	4.6, 4.6	9.5 (*n* = 5)

^a^Mean values obtained for all mice in the group regardless of clinical condition

^b^Mean values obtained for mice observed to have observable or pronounced clinical signs

^c^Values obtained from brains excised on the day mice succumbed to disease (i.e. day 3–4 post challenge)

**Fig. 2:**
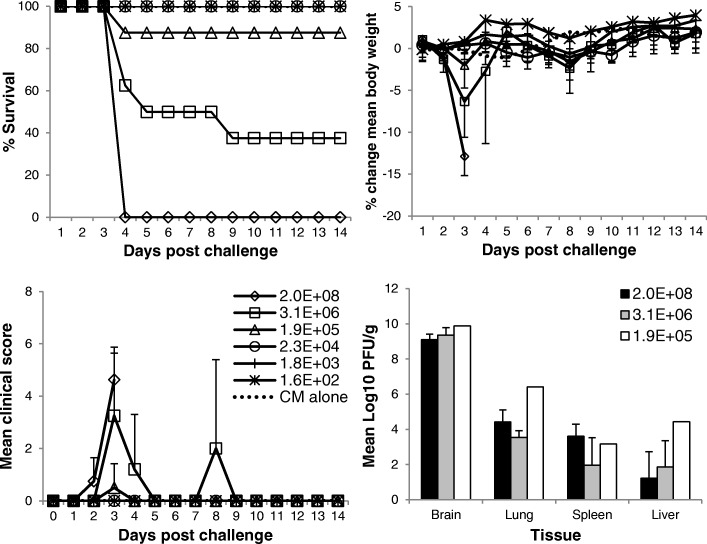
Experimental outcomes in Balb/c mice exposed by the aerosol route to a dose range of EEEV Pe-6, or challenge media controls (CM alone). Top left panel; percentage survival post- challenge. Top right panel; percentage change in body weight (from weight taken on day 0 prior to challenge). Bottom left panel; mean clinical scores. Bottom right panel; mean viral titre in tissues of animals that succumbed to disease 3–4 days post-challenge. The legends refer to the titres of input virus (pfu/ml). Data points include mice that were free of clinical signs. Error bars indicate 95% confidence interval. *n* = 8 except for the bottom right panel, where *n* = 1–5

Survival was observed to be dose dependent. An input virus challenge dose of 2 × 10^8^ pfu/ml resulted in 0% survival, and ≤ 2.3 × 10^4^ pfu/ml resulted in 100% survival. Calculation of the MLD using the formula of Reed and Muench provides an MLD for EEEV Pe-6 of 145 pfu/mouse presented dose, when challenged nose-only, by the aerosol route. An increase in mortality corresponded with an increase in clinical scores and weight loss (Table 1, Fig. 2). Mice that received a less than lethal challenge dose did not show any clinical signs of disease. The survival of these mice is likely to be a result of an appropriate and sufficient immune response preventing the virus from gaining access to the central nervous system (CNS) or by reducing viral loads in tissues. The immunological profile was not assessed in this study although it would provide an understanding of this hypothesis. The mean time to death (MTTD) was ~ 3 days post challenge regardless of presented challenge dose. The MTTD of mice challenged with 4.3 × 10^2^ pfu/mouse presented challenge dose is 4.2. However, this is distorted by an outlier mouse from this group that succumbed to infection at 8 days post challenge. The remaining mortalities from this group had a MTTD of 3.2 days. This single outlier animal was a peculiarity, in that it was free from any clinical signs or substantial weight loss until abruptly and rapidly presenting on day 8 post challenge, with observable and then pronounced clinical signs. All other cage mates of this individual animal had succumbed to infection by day 3–4 post challenge, with expected clinical features. The possibility that this mouse may not have been infected as a result of the aerosol exposure but became infected via transmission from cage mates would appear unlikely, as a number of other animals that survived aerosol exposure where cage mates succumbed did not exhibit any indicators of disease (clinical score, weight loss). Additionally, there is no evidence of person-person transmission in humans for EEEV (or WEEV and VEEV).

The disease course was rapid and aggressive, particularly in animals challenged with the highest doses of virus. In total 6.3% (3/48) were found to have succumbed to infection, 22.9% (11/48) were culled on welfare grounds, and the remaining animals were free of clinical signs during the 14 day observation period. Clinical signs included pronounced piloerection, hunched posture, altered behaviours and mobility issues (lateral/ventral recumbency, inability to move to reach food or water). Altered behaviour included fixed gaze, pronounced reduction in co-ordinated motor control and no reaction to stimuli (no attempt to move, hide, close eyes despite provocation). Mice were not observed to have any excitable behaviours, did not display other common altered behaviours such as spinning, as has been observed in other aerosol models of alphavirus disease, nor were their eyes observed to be affected. Mice exposed to ≤24 pfu/mouse presented dose (1.9 × 10^5^ pfu/ml input virus) did not present with any clinical signs and maintained a typical weight profile (with the exception of a single animal exposed to 24 pfu/mouse). CM alone (no virus) challenged mice also maintained a typical weight profile and were free of clinical signs throughout the observation period. The mean viral load observed in specific tissues of representative mice that succumbed to disease was also determined, demonstrating consistently high viral titres regardless of challenge dose (Fig. 2).

### Pathogenesis of EEEV Pe-6 infection

**Fig. 3:**
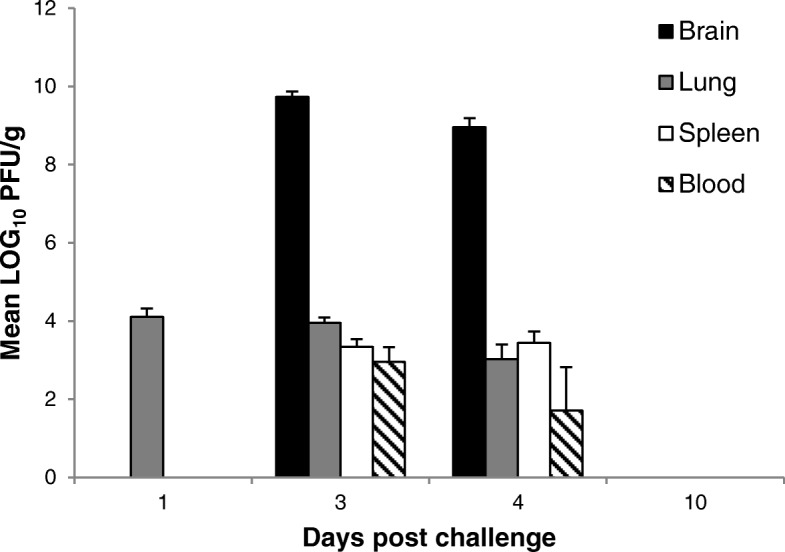
Mean viral load of EEEV Pe-6 in Balb/c mice exposed to 5.5 x MLD by the aerosol route. Error bars indicate 95% confidence interval, *n* = 2–22

Adult Balb/c mice were challenged with a mean calculated presented dose of 5.5 × MLD and, at scheduled time-points post challenge, were sacrificed to determine pathogenesis of disease. Blood was obtained by cardiac puncture prior to cull, and key tissues were excised to determine viral load. During the course of this experiment, 5% (3/60) of EEEV infected mice succumbed to disease 3 days post challenge, 30% (18/60) were culled on welfare grounds (3–4 days post challenge), and the remainder were culled to meet pre-determined time-points. Virus was first detected in the lung at 1 day post challenge, establishing a peak mean titre of 4.1 log_10_ pfu/g (Fig. 3), although overt clinical signs of disease were absent at this time. Virus was not detected in the brain, spleen or blood 1 day post challenge. Positive brain and spleen samples were observed 3 and 4 days post challenge with peak mean titres of 9.74 Log_10_ pfu/g in the brain and 3.44 Log_10_ pfu/g in the spleen. Viraemia was also observed at this time, establishing a peak mean titre of 2.95 Log_10_ pfu/ml of blood, although two individual mice were negative for EEEV in the blood despite being positive in all other tissue types tested. Peak titres in the brain and spleen coincided with pronounced clinical signs of disease with the exception of a single mouse culled 3 days post challenge to meet a scheduled time-point, which was free from *pronounced* clinical signs yet positive for EEEV in all tissue types tested. Mice sacrificed 10 days post challenge were negative for virus in all tissue types tested, and were free of clinical signs throughout the study. The viral load of tissues and blood at time-points outside of those presented here is not known, and although the plaque assay has a low limit of detection (10 pfu/ml), it is possible that samples presented as negative, contained viable virus.

A representative group of mice from each scheduled time-point was preserved by immersion in 10% NBF for subsequent histopathological analysis. Whole carcasses were processed and prepared for histopathology as described. CM only (no virus) challenged animals were sacrificed 1 day post challenge, and were observed to be within normal limits, with the absence of any marked histopathological findings, for all tissue types examined. All EEEV challenged animals were compared to this control group. IHC techniques were employed to specifically identify EEEV antigen, although non-neuronal tissues were excluded from analysis as high background and non-specific staining was observed in epithelial cell cytoplasm and nuclei, presumably from the presence of endogenous antibody outside neuronal tissues and/or the cross linking of OMap anti-mouse HRP multimer to mouse antibodies.

**Fig. 4:**
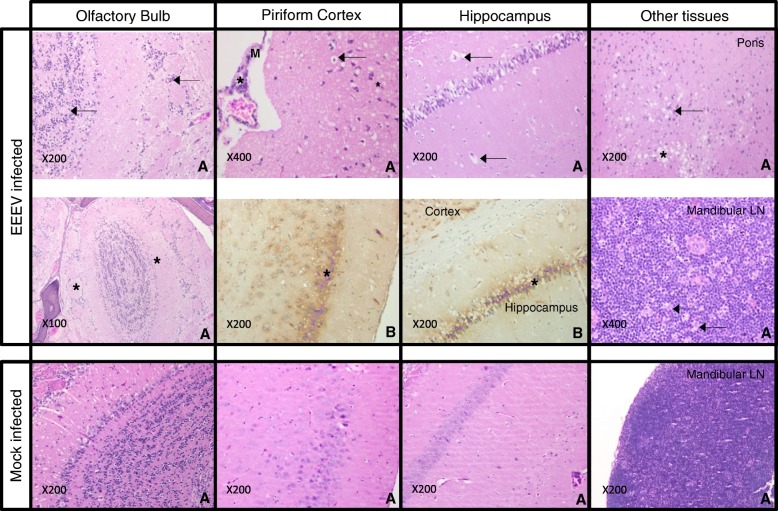
Groups of Balb/c mice were challenged with EEEV Pe-6 by the aerosol route and culled at pre-determined time-points, culled on welfare grounds, or were found dead. Histopathology (**a**) and immuno-histochemical demonstration of EEEV virus (**b**) images were obtained from multiple animals, represented here by two mice that illustrate key histopathological features of disease. Mice exposed to CM alone (mock infected) were found to have neuronal tissues within normal limits, with distinct nuclei in well-defined neuronal layers, as well as clear white matter tracts and nerves that were strongly eosinophilic in the olfactory bulb. Neurons were not vacuolated and perivascular spaces were also within normal limits for these tissues 1 day post-challenge. Mice exposed to EEEV presented with severe meningoencephalitis with cell death and degenerative changes in neurons and leukocytes in the encephalon, with marked rarefaction and/or oedema of the tissue by day 3 post-challenge. In the olfactory bulb, there was an abundance of shrunken neurons and cell debris (arrows), and rarefaction of the neural tissue (*). In the piriform cortex, there was abundant pyknotic and karyorrhectic debris in the vessel wall (*) and in the CNS (arrows), as well as considerable changes in the perivascular spaces of the meninges (M). Contralateral piriform cortex sections demonstrate EEEV-specific labelling in neurons (*). The hippocampus had marked vacuolation (*) and clear expansion of perivascular spaces (arrows), with diffuse EEEV-specific labelling of neuronal populations (*). Other tissues with diffuse EEEV-specific labelling included the cortex, hippocampus and thalamus. In the pons region of the brain there was a focal area of spongiosis/vacuolation (*), as well as neuronal degeneration, necrosis and abundant pyknotic and karyorrhectic debris (arrows). The mandibular lymph node (LN) displayed numerous tingible body macrophages (arrows) and had a marked increase in apoptopic bodies

At 1 day post-challenge there were no prominent histopathological findings in any of the EEEV infected animals. By day 3 post-challenge however, all of the animals examined presented with severe meningoencephalitis, with notable cell death and degenerative changes in neurons and leukocytes in the encephalon, as well as oedema and a marked reduction in cell density (rarefaction) (Fig. 4). Enlarged perivascular spaces were observed in the meninges of the piriform cortex, with abundant pyknotic and karyorrhectic debris in the vessel walls and in the central nervous system, indicative of substantial cell degeneration and apoptosis. Additionally, there was marked vacuolation of hippocampal neurons and again, expansion of perivascular spaces. Lesions were mostly bilateral (affecting both brain hemispheres, which are independently connected to the left and right olfactory epithelium), although there was variability between individuals, with one side of the brain more severely affected in most cases, and a small number of animals with only unilateral presentation. In nearly all cases, only the cortex and caudate putamen were affected (rostral regions), although in one animal lesions reached as far back as the thalamus and pons (Fig. 4). Rostroventral areas tended to be more severely affected. Lesions in the olfactory bulb matched the presence of lesions in the affected brain hemisphere. For example, animals with unilateral lesions were affected in only one (corresponding) olfactory bulb. Rare vacuolated cells were also apparent, as well as an increase in the presence of cell debris in the olfactory epithelium, which is likely to be associated with an increase in apoptosis, attributable to viral infection. Reactive changes were observed in the mandibular lymph node at this time too, including numerous tingible body macrophages, and an increase in apoptopic bodies. At 4 days post-challenge animals remained severely affected with meningoencephalitis, displaying comparable distribution, extension and severity of lesions as those animals examined at 3 days post-challenge, although only unilateral presentation was observed at this time-point. Lesions were located almost exclusively in the cortex and caudate putamen (rostral regions), with only a single animal found to have lesions in more caudal structures. Notable changes were again observed in the olfactory epithelium, with marked rarefaction when compared to the control group. No marked histopathological changes were observed in animals culled 10 days post-challenge. It is possible that these animals were able to mount an appropriate immune response to prevent or reduce the severity of neuroinvasion, or were not exposed to sufficient viral titre to cause infection as there was a lack of histopathological findings in these animals. Cage mates exposed simultaneously to the same EEEV aerosol exposure presented with pronounced clinical signs of disease and succumbed to infection, and it is therefore unlikely that insufficient virus was present in the presented aerosol. The immune response in these animals is the likely cause of survival, although immunological responses were not studied.

The use of a mouse monoclonal antibody for IHC caused considerable cross reactivity with cells and plasma, preventing effective analysis of viral antigen in non-neural tissues. There was also a lack of immuno-labelling in the olfactory bulbs of animals positive for EEEV in rostral regions. This was potentially an artefact of the decalcification process, specifically affecting the antigenicity of the tissues. Labelling was observed as fine granules in neuronal cytoplasm/perykarion or outlining neurons, and in neuropil. All animals that succumbed to disease were positive by IHC, with a diffuse distribution throughout the brain, and affecting most areas (apart from the olfactory bulb and cerebellum) as early as 3 days post challenge. Immuno-labelling extended further caudally than the lesions observed during histopathological examination. The highest concentration of immuno-labelled cells was found in the rostroventral areas of the brain and cortex, including the piriform cortex, with fewer positive cells in the most caudal regions (pons and medulla). Some animals presented with unilateral distribution of lesions in the brain, yet virus antigen was detected by IHC in both hemispheres. The acute clinical course of EEEV disease may be a contributory factor for the limited lesion and virus distributions. In a similar study, WEEV infected mice presented with qualitatively similar histopathological findings, yet were observed to have lesions beyond cortical structures and the caudate putamen [6]. This may be due to the increased mean time to death of WEEV-infected mice exposed to virus by the aerosol route.

## Discussion

In the event of a bio-warfare scenario or an accidental laboratory exposure where people may be exposed by the aerosol route to EEEV, it is important to understand the particular hazard of EEEV infection by this route. Human cases of aerosol infection with alphaviruses are rare, although a report by Hanson et al. [9] describes a mortality rate of 40% (2/5) when laboratory personnel were accidentally exposed to WEEV aerosols. Here we show that exposure to EEEV Pe-6 by the aerosol route was lethal in adult female Balb/c mice, with an acute disease course (mice succumb 3–4 days post challenge), progressively severe clinical signs of disease and 100% mortality when mice were challenged with 2.8 × 10^3^ pfu/mouse presented dose. The MLD in this model of EEEV Pe-6 was calculated as 145 pfu/mouse presented dose by the aerosol route. Other researchers have reported the use of small animal models to determine susceptibility and lethality of EEEV (strain NJ1959), where the virulence of whole-body aerosol exposure to EEEV was observed to be greater in mice than in guinea pigs with an LD_50_ of 620 and 11,000 pfu/animal presented dose respectively [17]. This and other calculations of mouse aerosol LD_50_ [4, 5] are similar to the MLD observed in our study, where mice were exposed in a nose-only exposure protocol. Honnold *et al* [7] have characterised EEEV disease in Balb/c mice infected with the virulent FL93–939 strain by three different routes of infection, including aerosol. Virus was detected as early as 6 h post infection, although the difference in the age of Balb/c mice (8–10 weeks old), and more likely, the differences in aerosolisation protocol (whole-body), challenge preparation (sucrose purified), concentration (100 × LD_50_) and strain (FL93–939), may account for the later onset of neuro-invasion with 5.5 × MLD EEEV strain Pe-6. Virus distribution and load was comparable with Honnold et al. I, [7] in the brain, although unsurprisingly a generally higher viral load was observed in the lung, spleen and blood of mice exposed to 100 × LD_50_ EEEV FL93–939 than described here. Exposure of marmosets to EEEV by the aerosol route culminates in a very similar disease course, where clinical signs were observed from 3 to 4 days post challenge, and included ruffled fur, lack of grooming and progressively, loss of balance, subdued behaviours, tremors and death in animals exposed to high doses of the virus (> 1 × 10^3^ pfu inhaled dose) [12]. Unlike rodent models, the higher the challenge dose of virus the lower the MTTD and the earlier severe clinical signs were observed in marmosets, with a dose-dependent MTTD range of 4–19 days for animals that succumbed to disease. This has also been observed in cynomolgus macaques exposed to EEEV by the aerosol route [13] and is an important difference between small rodents and non-human primates.

Acute clinical signs and weight loss were associated with all animals that either succumbed to disease or were culled on welfare grounds. Weight loss across all groups was < 15% of initial body weight. This was statistically significant in animals receiving 2.8 × 10^3^ pfu/mouse (*P* = 1.5 × 10^− 7^, ANOVA). However even at a dose of 4.3 × 10^2^ pfu/mouse, where 5/8 animals succumbed, weight loss was not statistically significant (*P* = 0.09, ANOVA). At all lower challenge doses, no significant weight loss was observed (*P* ≥ 0.27). Consequently, weight loss was not an appropriate parameter on which to base a humane endpoint. Animals that did not present with clinical signs and did not experience substantial weight loss, were free of viable virus in all tissue types examined at 14 days post challenge. Although development of clinical signs was progressive, the onset of severe clinical signs was abrupt. The maximum observed weight loss did not exceed ~ 13% of original body weight in all mice that succumbed to infection, and the highest mean clinical score in the highest challenge dose was relatively low (mean 4.6). This is despite the fact that mice that succumbed to disease and were discovered dead at a routine check (as opposed to reaching a humane endpoint and being culled) were allocated the maximum score (eight) recorded for culled mice, for the day of discovery. The highest mean clinical scores of WEEV mice infected by the aerosol route, using the same clinical scoring system, were typically ~ 10 [6]. The relatively low clinical scores for groups with 100% mortality for EEEV is reflective of less severe clinical signs in the lead up to a sudden, severe decline in health. For example, a mouse presenting as immobile and unable to reach food and water, scored 2 only, and would be culled despite the absence of other clinical signs. Tremors/shaking were not a general feature of this study (only observed in a single animal exposed to the highest dose of virus). Animals succumbed to infection rapidly after the onset of *any* clinical signs within 24 h.

The presentation of overt clinical signs coincides with peak viral titres in the tissues examined. The overt pronounced altered behaviour (fixed gaze, reduction in co-ordinated motor control, inability to respond to stimulus) also coincides with high viral titres of EEEV in the tissues examined at 3 days post challenge. Such an established infection is the likely cause of this altered behaviour although this may be tissue-dependent and/or viral load-dependent, as 6/16 mice processed for histology and IHC at this time that exhibited only minor clinical signs (observed to have ruffled fur, with or without hunched posture) were found to have bilateral lesions, although only extending to the most rostral and ventral cortex regions of the brain. These mice were also positive for EEEV antigen with diffuse labelling in the rostroventral cortex (negative in the hippocampus, hypothalamus, thalamus, pons medulla and cerebellum). The remaining mice (10/16) processed at this time exhibited pronounced clinical signs, and had very similar histopathological findings as well as an extension of the infection out to the hippocampus, hypothalamus, thalamus, medulla and pons (varying degrees in different animals), including two mice with brain haemorrhage. It is possible that this viral load in the brain presents a critical threshold past which clinical signs abruptly develop. These histopathological findings are in close agreement with Honnold et al. II [8], where there is overwhelming infection throughout the brains of mice exposed by the aerosol route by day 3 post challenge, following a pathological trail from the olfactory bulbs to the piriform cortex (expanded perivascular spaces and inflammatory changes), with rostral to caudal spread, extending out to the hippocampus, the cortex (lesions), cerebellum and pons (spongiosis) by day 4 post challenge. The presence and severity of lesions within the individual olfactory bulb relates to the severity and spread of infection in the corresponding brain hemisphere. The ability of this virus to gain rapid access to the brain when introduced in an aerosol results in a devastating meningoencephalitis with cell death, degenerative changes in neurons and leukocytes in the encephalon, as well as marked rarefaction of the tissues. Because our study uses a much lower MLD challenge dose than other studies, not all animals succumbed, and we are able to show evidence suggesting the abrupt onset of severe clinical signs is linked to a critical threshold of viral load in the brain.

EEEV is considered the most deadly of the three major encephalitic alphaviruses, with a high mortality rate of 36–75% [2]. In humans, during natural outbreaks of the virus, clinical features may include sudden onset of fever, headache, chills, arthralgia and malaise. A proportion of those infected may go on to experience neurological involvement manifesting as fever, headache, drowsiness, restlessness, irritability, confusion, vomiting, cyanosis and seizures, leading to coma and possibly death. In this mouse model of aerosol induced infection, the non-specific clinical features present abruptly as a combination of clinical signs; ruffled fur, hunched posture, a change in respiratory rate, and weight loss. This promptly leads to pronounced changes in mobility and behavior (e.g. lateral/ventral recumbency, loss of balance, fixed gaze), although tremors/seizures were not observed. The studies of Honnold et al. I [7] also describe focal muscle twitching and on rare occasions seizures, and Roy et al. [17] also describe a generalized flaccid paralysis in Balb/c mice. It is possible that twitching and seizures may have occurred during our studies but were not witnessed. We did observe twitching in a single animal that received the highest presented dose of EEEV (2.8 × 10^3^ pfu/mouse), which may be due to our earlier MTTD (3 days vs. 5.8 days [17]) or our use of lower challenge doses (100 × LD_50_ vs. ~ 20 × MLD as highest dose in our study).

An aerosol challenge of EEEV Pe-6 by the aerosol route recapitulates the most severe clinical features observed in humans, where animals displayed overt clinical signs, including neurological signs, and ultimately death. The mortality rate is higher in mice than in naturally occurring epizootic episodes, and this is likely, among others factors, to be attributed to the route of infection. Severe neurological signs precede death, and these appear to be linked to a threshold level of virus replication in the brain. Consequently, effective treatments for EEEV encephalitis are likely to require early administration to prevent infection of the brain in zoonotic cases, and the ability to directly or indirectly cross the blood brain barrier to halt replication in the brain in cases of aerosol infection. The use of antibodies as medical countermeasures against aerosol induced VEEV requires delivery of the antibody within a specific window to elicit protection, typically within 48 h [18–22]. To date, there have been no published reports regarding the protective efficacy of antibodies directed against EEEV when challenged by the aerosol route. Small molecule antiviral compounds may also have utility as a medical countermeasure. Lundberg et al. [23] screened a number of compounds already approved for other indications in vitro, demonstrating inhibition of replication for both VEEV and WEEV when treated with sorafenib (carcinoma therapeutic). There is no published assessment of sorafenib in vivo against VEEV or WEEV, and there remain no post exposure putative candidate antivirals for use against EEEV.

In a prophylactic setting, a number of candidate encephalitic alphavirus vaccines have been assessed in vivo [24–27], including a number of Phase I clinical trials [28, 29]. To date none of the candidate vaccines have progressed to Phase II clinical studies. Further work is necessary to better understand the pathophysiological, neurological and immunological facets of aerosol induced encephalitic alphaviral disease. A lack of human data necessitates the use of robust in vivo models, especially for EEEV, as EEEV is the least well studied or understood of the three encephalitic New World alphaviruses.

## Conclusions

This report describes the susceptibility of adult Balb/c mice to the highly pathogenic EEEV Pe-6 by the aerosol route and a determination of the associated MLD. The pathogenesis of disease is also detailed, describing viral load and distribution, as well as histopathology within neuronal tissues. Establishing this model in mice provides an important tool for the robust assessment of candidate medical countermeasures against a lethal inhalational challenge. Such assessments may provide an evidence base on which to undertake development of licensed medical countermeasures for this highly pathogenic, aggressive, neuro-invasive virus.

## Abbreviations

BW: Biological warfare
CDC: Centre for Disease Control
CM: Challenge medium
CNS: Central nervous system
EEEV: Eastern equine encephalitis virus
FDA: Food and Drug Administration
IHC: Immuno-histochemistry
LD_50_: Dose of virus required to be lethal in 50% of a population
MLD: Median lethal dose
MM: Maintenance medium
MTTD: Mean time to death
PFU: Plaque forming unit
VEEV: Venezuelan equine encephalitis virus
WEEV: Western equine encephalitis virus
